# Notable epigenetic role of hyperhomocysteinemia in atherogenesis

**DOI:** 10.1186/1476-511X-13-134

**Published:** 2014-08-21

**Authors:** Shuyu Zhou, Zhizhong Zhang, Gelin Xu

**Affiliations:** Department of Neurology, Jinling Hospital, Nanjing University School of Medicine, 305 East Zhongshan Road, Nanjing, 210002 Jiangsu Province China

**Keywords:** Atherosclerosis, Epigenetics, DNA methylation, Hyperhomocysteinemia

## Abstract

Atherosclerosis is associated with multiple genetic and modifiable risk factors. There is an increasing body of evidences to indicate that epigenetic mechanisms also play an essential role in atherogenesis by influencing gene expression. Homocysteine is a sulfur-containing amino acid formed during methionine metabolism. Elevated plasma level of homocysteine is generally termed as hyperhomocysteinemia. As a potential risk factor for cardiovascular diseases, hyperhomocysteinemia may initiate or motivate atherogenesis by modification of DNA methylation. The underlying epigenetic mechanism is still unclear with controversial findings. This review focuses on epigenetic involvement and mechanisms of hyperhomocysteinemia in atherogenesis. Considering the potential beneficial effects of anti-homocysteinemia treatments in preventing atherosclerosis, further studies on the role of hyperhomocysteinemia in atherogenesis are warranted.

## Introduction

Epigenetics is defined as changes in phenotype and gene expression that occur without alterations of DNA sequence [1]. By means of gene-environment interactions, epigenetic mechanisms can be acquired and/or heritable throughout lifespan. There are three major epigenetic types: (1) DNA methylation, (2) histone modification, and (3) noncoding RNA regulation. DNA methylation, occurred in cytosine residues of CpG dinucleotides, is mediated by DNA methyltransferases (DNMTs). During evolution, the CpG dinucleotides have been progressively eliminated from the genome and are present at only 5% to 10% of its predicted frequency. Cytosine methylation appears to play a major role in this process because of the high susceptibility of 5-methyl cytosine to undergo spontaneous deamination to yield thymine [2]. DNA methylation is the most well-known epigenetic mechanism, and plays a critical role in the regulation of global and specific gene expression [3]. Intriguingly, recent evidences identified some allele-specific DNA methyation (ASM) [4–6] and methlylation-associated loci (meQTLs) [7]. These novel concepts, for the first time, associate genetic variations with epigenetic changes. The interaction between genetic variants and DNA methylation also emphasize the need for an integrated study [8].

Atherosclerosis is a chronic inflammatory disease of large or intermediate arteries. It is pathologically characterized by infiltration of lipid particles, endothelial activation, macrophage infiltration and foam cell formation. The foam cell formation, known as “fatty streak”, followed by smooth muscle migration and proliferation, and extracellular matrix deposition usually resulted in the formation of an atherosclerotic plaque, which may eventually rupture and cause a cardiovascular event, such as stroke or myocardial infarction.

The epigenetic impacts on cardiovascular diseases (CVD) have garnered considerable research interests since the initial suggestion of epigenetics in 1999 [9]. Atherogenesis has been proposed as a result, at least partly, of diet-induced DNA methylation. Although genome-wide association study (GWAS) indentified a number of single nucleotide polymorphisms (SNPs) associated with CVD, most of these SNPs have not been previously implicated in the pathogenesis of atherosclerosis and have modest biological plausibility [10]. It seems that the GWAS identified genetic discrepancies only account for a small fraction of heritability of atherosclerosis. Hence, epigenetics is emerging in the “post-GWAS” era as the next clue in probing the mechanisms of atherogenesis. It is expected to provide the previously missed link among gene, environment and disease.

Hyperhomocysteinemia (HHcy) is an established risk factor for atherosclerosis [11–14]. HHcy can increase oxidative stress, activate inflammatory, and promote vascular smooth muscle cells (VSMCs) proliferation, all of which may result in initiation of atherosclerosis [15, 16]. Since homocysteine (Hcy) is a key component of methionine recycle system, plasma Hcy level may be associated with DNA methylation and other epigenetic modification. Thus, a better understanding of the role of Hcy metabolism as a part of one-carbon metabolism is essential and may provide useful information in establishing efficacious strategies for preventing and treating atherosclerotic diseases.

### Homocysteine

**Figure 1:**
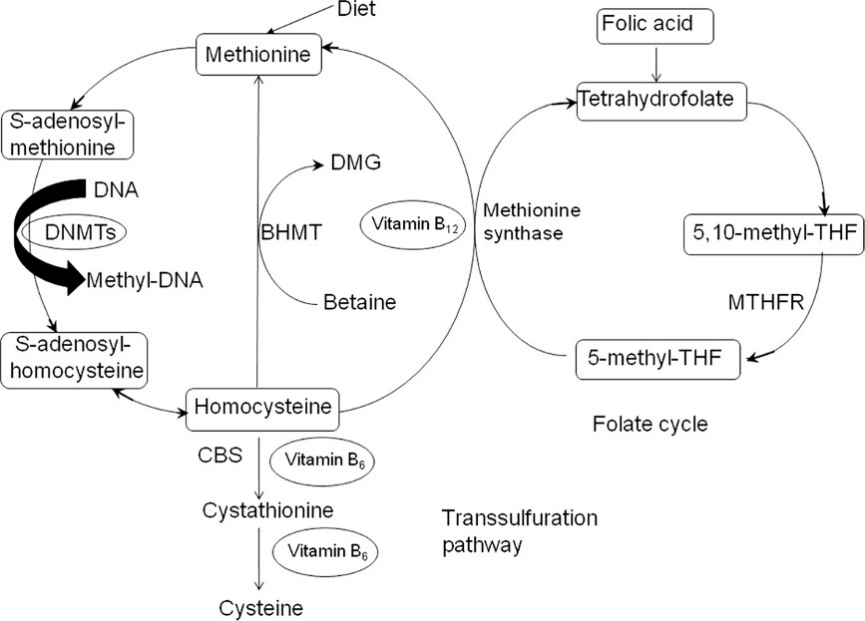
**Methionine recycle system and homocysteine metabolism.** BHMT: Betaine-homocysteine methyltransferase, DMG: Dimethylglycine, THF: tetrahydrofolate, MTHFR: 5,10-methyltetrahydrofolate reductase, CBS: cystathionine β-synthase.

Homocysteine (Hcy) is a sulfur-containing amino acid derived from methionine after demethylation via two intermediate compounds, S-adenosylmethionine (SAM) and S-adenosylhomocysteine (SAH) [17]. Methionine is an essential amino acid acquired mostly from the methionine recycle system and partly from the diet (Figure 1). It can combine with adenosine triphosphate to yield SAM, which is the most important donor to methyl group in human body. With the transfer of a methyl group, SAM is converted to SAH and the SAM/SAH ratio may serve as an indicator for intra-cellular methylation capacity [18–20]. Most SAM-dependent methyltransferases, including DNA methyltransferases (DNMTs), can be inhibited by SAH which has a higher affinity with methyltransferases than SAM [21]. SAH can be further hydrolyzed to Hcy and adenosine. This reaction is reversible with a thermodynamic equilibrium that strongly favors SAH synthesis rather than hydrolysis [15].

Hcy is metabolized in vivo via two pathways: remethylation or transsulfuration. In remethylation pathway, Hcy is first transformed to methionine by the addition of a methyl group from 5-methyltetrahydrofolate or betaine. 5-methyltetrahydrofolate is a product of the conversion of folic acid to 5,10-methyltetrahydrofolate and finally metabolized to 5-methyltetrahydrofolate by enzyme 5,10-methyltetrahydrofolate reductase (MTHFR). In almost all tissue types, the cofactor vitamin B_12_ participates in the remethylation with 5-methyltetrahydrofolate, whereas the reaction with betaine is restricted to liver, and is independent of vitamin B_12_. In the transsulfuration pathway, Hcy is converted to cystathionine by cystathionine β-synthase (CBS) and finally to cysteine with vitamin B_6_ as a cofactor [22].

### Hyperhomocysteinemia

Plasma Hcy levels usually vary between 5 and 15 μmol/L in healthy adults. According to fasting plasma Hcy levels, hyperhomocysteinemia (HHcy) may be classified as moderate (15-30 μmol/L), intermediate (31-100 μmol/L) and severe (>100 μmol/L) [14, 23]. HHcy originates from a deviation in the methionine-homocysteine metabolism including disturbances of enzymes, vitamin deficiencies and other factors [14, 23, 24], as shown in Table 1.

**Table 1:** **Causes of hyperhomocysteinemia**

Genetic defects	Vitamin deficiencies	Other factors
Cystathionine β-synthase deficiency;	Lack of folic acid;	Age;
	Lack of vitamin B_6_;	Male sex;
5,10-methyltetrahydrofolate reductase deficiency;	Lack of vitamin B_12_	Lifestyle factors (smoking, coffee, alcohol abuse);
Methionine synthase deficiency;		Chronic renal insufficiency;
		Hepatic dysfunction;
Genetic defects in the vitamin B_12_ metabolism		Systemic lupus erythematosus;
		Cancers;
		End stage diabetes mellitus
		Hypothyroidism et al.

Moderate HHcy (15-30 μmol/L) usually reflects impaired pathway of remethylation. The possible causes include deficiency of folic acid, vitamin B_12_ or dysfunction of MTHFR. A point mutation of amino acid 677 (677 C → T) in MTHFR gene can causes alanine-valine substitution and is associated with reduced enzyme activity of MTHFR. This is the commonest form of genetic HHcy [25]. Severe HHcy (>100 μmol/L) may be caused by deficiency of homozygote CBS, homozygote thermo-stable MTHFR, or enzymes catalyzing vitamin B_12_ metabolism. Abnormal increase of plasma Hcy (>15 μmol/L) after a methionine load (100 mg/kg) may reflect impaired Hcy transsulfuration due to deficiency of heterozygous CBS or vitamin B_6_ [22].

HHcy is observed in approximately 5% of the general population and is associated with increased risk of CVD, autoimmune disorders, birth defects, diabetic mellitus, renal diseases, osteoporosis, neuropsychiatric disorders and cancer [26]. Several studies have identified moderate HHcy as an independent risk factor for atherosclerotic diseases [27].

### Hyperhomocysteinemia and DNA methylation

In the methionine recycle system, SAH hydrolyzes to Hcy and adenosine. This reaction is reversible, hence, elevated Hcy level may induce SAH synthesis. The increased SAH can, via a negative feedback, inhibit SAM-dependent methyltransferases, such as DNMTs. DNMTs mediate DNA methylation by transferring methyl groups from SAM to cytosine residues in a CpG dinucleotide context. Thus, dysfunction of Hcy metabolic pathways may result in DNA hypomethylation. There are increasing evidences to indicate that HHcy may be associated with DNA methylation levels in vivo. The pioneering work of Yi and colleagues [28] in 2000 showed that plasma total Hcy level (in healthy subjects) were associated with plasma SAH, lymphocyte SAH and lymphocyte DNA hypomethylation levels. In cardiovascular patients with concomitant HHcy, simultaneous elevation of plasma SAH [29] and disturbance of DNA methylation [30] were observed. This association was confirmed in animal studies [31, 32].

In human somatic cells, methylated cytosine accounts for about 1% of total DNA bases and affects 70-80% of all CpG dinucleotides in the genome [33]. Unmethylated CpGs are grouped in clusters called “CpG islands” that are present in the 5′ regulatory regions of many human genes [34]. DNA methylation may influence the transcription of genes in two ways. The presence of methyl group at a specific CpG dinucleotide site may directly prevent DNA from recognizing and binding to transcription factors [35]. In other instance, methylated DNA may be bound by proteins known as methyl-CpG-binding domain proteins (MBDs). These MBDs can directly repress transcription, prevent the binding of activating transcription factors, or recruit enzymes that catalyze histone posttranslational modifications and chromatin-remodeling complexes that alter the structure of chromatin and actively promote transcriptional repression [36]. In general, DNA methylation is associated with low gene activity. Global or specific DNA methylation may contribute to altered gene expression, and may lead to vascular damage.

### Hyperhomocysteinemia, DNA methylation and atherogenesis

Atherosclerosis is a dynamic process involving several cell types such as monocytes, endothelial cells, and smooth muscle cells (SMCs). A chronic inflammatory response with infiltration of macrophages and T-cells along with endothelial dysfunction is also prominent in the pathogenesis of plaque formation. In response to inflammation or injury, production of ROS is enhanced in vascular cells. These changes all contribute to the initiation and progress of atherosclerosis. There has been a variety of evidences to indicate that epigenetic changes play an important role in atherogenesis beside genetic and environment factors [37–41].

SMCs play a unique role in the development of atherosclerosis. Hypomethylation has been observed in proliferated VSMCs from advanced human atherosclerotic plaques, and from atherosclerotic lesions in mouse and rabbits [31, 42, 43]. Hypomethylation is correlated with increased transcriptional activity that may affect cellular proliferation and gene expression. Using VSMCs in culture, Yideng et al. [44] observed hypomethylation of LINE-1 and Alu elements in medium with high Hcy concentration. Their results indicated that HHcy may increase SAH and decrease SAM concentrations, change SAH hydrolase expression in RNA and protein levels, and enhance activity of DNA methyltransferase [45]. Researchers concluded that the dissimilar detrimental effects of Hcy in various concentrations may be functioned by different mechanisms. Mild or moderate HHcy may influence gene expression mainly through the interference of transferring methyl-group metabolism. However, severe HHcy may educe more injurious effects by increasing oxidative stress, promoting apoptosis and inflammation. HHcy induced SAH elevation can promote VSMC proliferation and migration through an oxidative stress-dependent activation of the ERK1/2 pathway, which in turn can facilitate atherogenesis in apolipoprotein E (ApoE)-deficient mice [46].

Estrogen receptors (ERs) are expressed in SMCs and endothelial cells in coronary artery, and may play an important role in preventing atherosclerosis [47]. The protective effects of estrogens against oxidative stress may mediate by ERα. Decreased ERα level can deteriorate atherosclerosis in men [48]. According to the study with VSMCs from human umbilical vein, Hcy can induce de novo methylation in the promoter region of the ERα gene, and subsequently down-regulate the expressions of ERα mRNA [49]. Hypermethylation of CpG islands located in promoter region of ERα gene is positively correlated with the plasma Hcy level, and facilitate the initiation and development of atherosclerosis.

Jamaluddin et al. [50] revealed that HHcy may exert highly specific inhibitory effects on cyclin A transcription and endothelial cells (ECs) growth through a hypomethylation related mechanism which blocks cell cycle progression and endothelium regeneration. Cyclin A suppression has been proposed as a possible mechanism for inhibiting EC growth, and therefore, may increase the risk of CVD. Furthermore, HHcy-mediated dysfunction of endothelial nitric oxide (NO) system is an important mechanism for atherosclerotic pathogenesis [51]. Dimethylarginine dimethylaminohydrolase (DDAH) is the key enzyme for degrading asymmetric dimethylarginine (ADMA), which is an endogenous inhibitor of endothelial nitric oxide synthase (eNOS). Using human umbilical vein endothelial cells (HUVECs), Zhang and colleagues observed that mildly increased Hcy concentration (10 and 30 μmol/L) may induce hypomethylation, while higher Hcy concentration (100 and 300 μmol/L) may induce hypermethylation in the promoter CpG island of DDAH2 gene [52]. The mRNA expression of DDAH2 increased in mildly increased concentration of Hcy, and decreased in higher concentration of Hcy correspondingly. The inhibition of DDAH2 activity, the increase of ADMA concentration, the reduction of eNOS activity and the decrease of NO production were all consistently relevant to the alteration of Hcy concentration. HHcy may influence the methylation of DDAH2 gene, and indirectly influence the function of NO system. This process may be an important pathway for the development of atherosclerosis involving NO system. Moreover, a recent study suggested that hypermethylation of DDAH2 contributes to apoptosis of ECs induced by Hcy [53]. DNA methylation inhibitor 5-azacytidine could attenuate the effect of Hcy on ECs.

In mutant mice deficient in MTHFR, global DNA hypomethylation was shown in both heterozygous and homozygous knockouts [54]. Abnormal lipid deposition was observed in the proximal aorta in elder heterozygotes and homozygotes, suggesting an atherogetic effect of HHcy. ApoE gene has been associated with atherosclerosis. Researchers found that clinically relevant Hcy level (100 mM) may increase the total cholesterol (TC), free cholesterol (FC), and cholesteryl ester (CE) levels, and decrease ApoE mRNA and protein expression levels in cultured human monocytes. All these effects may be caused by increased DNA methylation of ApoE [55]. Peroxisome proliferators-activated receptor α and γ (PPARα and γ), acted as lipid sensors and bound with high affinities to ligands of anti-atherosclerosis, were also observed concomitantly with hypermethylation in promoter induced by Hcy in monocytes [56]. Recently, Wang et al. [57] confirmed that DNA hypomethylation in promoter region of monocyte chemoattractant protein-1 (MCP-1) gene through NF-κB/DNMT1 may play a key role in the formation of atherosclerosis under HHcy condition in ApoE–deficient mice.

Cholesterol-loaded foam cells usually form the core of atherosclerotic lesions. ATP-binding cassette transporter A1 (ABCA1), which mediates the efflux of cellular cholesterol and phospholipids, is the rate-limiting step in lipid metabolism. Acyl-coenzyme A: cholesterol acyltransferase-1 (ACAT1) promotes accumulation of CE in macrophages, thereby resulting in the foam cell formation, a hallmark of early atherosclerotic plaque. In the study by Liang et al. [58], cultured monocyte-derived foam cells were incubated with clinical relevant concentrations of Hcy for 24 h. Number of foam cells and cholesterol level were increased, but the mRNA and protein expression of ABCA1 were decreased, while ACAT1 expression was increased in the presence of Hcy. The DNA methylation level of ABCA1 gene was increased whereas ACAT1 DNA methylation was decreased when Hcy concentrations were changed. Moreover, the results showed that DNMT activity and DNMT1 mRNA expression were increased by Hcy. It indicated that DNA methylation has the function to regulate the expression of ABCA1 and ACAT1 via DNMT. The results manifested that ABCA1 and ACAT1 DNA methylation induced by Hcy possibly play a potential role in ABCA1 and ACAT1 expression and the accumulation of cholesterol in foam cells.

DNA methylation may reflect altered immune or inflammatory responses during atherosclerosis among cell types [59]. Given the established roles of inflammation and leukocytes in atherosclerosis, peripheral blood leukocytes represent a biologically relevant cell type for cardiovascular studies. Castro et al. demonstrated that patients with vascular diseases have a disturbed global DNA methylation status, which was associated with plasma Hcy levels [30]. High blood Hcy levels correlate with DNA hypomethylation and atherosclerosis and can lead to a 35% reduction in the DNA methylation status of peripheral blood lymphocytes. In contrast to these findings, Sharma and coworkers observed a significant positive correlation of global DNA methylation with plasma Hcy levels in patients with coronary artery diseases [60]. They concluded that alteration in genomic DNA methylation and the association with CVD appear to be further accentuated by higher Hcy levels. After reviewed literatures regarding to 135 genes either modulating or modulated by Hcy, Sharma et al. concluded that elevated plasma Hcy may lead to atherosclerosis either by directly affecting lipid metabolism and transportation, or by oxidative stress and endoplasmic reticulum stress by decreasing the bioavailability of NO and modulating the levels of other metabolites, including SAM and SAH [61].

In conclusion, aberrant global DNA methylation is only an index of the potential for epigenetic dysregulation. An increasing number of factors that can modify the DNA methylation patterns have been identified. These include the rate of cell growth and DNA replication, chromatin accessibility, local availability of SAM, nutritional factors, duration and degree of the hyperhomocysteinemic state, inflammation, dyslipidemias, oxidative stress, and aging [62]. The relationship between HHcy and DNA global hypomethylation may be masked in the clinical setting owing to the presence of these confounders, thereby possibly explaining some contradictory and counterintuitive findings reported to date. Another important aspect to consider is that DNA methylation is unequally distributed throughout chromosomes of differentiated cells [63]. Thus, hypermethylated and hypomethylated regions can coexist in the genome. The global DNA methylation status need not correspond to the methylation status of specific genomic regions. In the presence of HHcy, more promoter regions of pro-atherogenic genes might be hypomethylated while anti-atherogenic genes hypermethylated. Thus pro-atherogenic genes gain more activity along with loss of protective function of anti-atherogenic genes which accelerate the process of atherogenesis ultimately.

### Hyperhomocysteinemia and histone modification

Nucleosomes are the basic units of chromatin and are composed of DNA wrapped around a protein octamer containing two molecules of each canonical histone (H2A, H2B, H3, and H4). Nucleosomes may be irregularly packed and fold into higher-order structures that occur in diverse regions of the genome during cell-fate specification or in distinct stages of the cell cycle. The arrangement of nucleosomes can be altered by covalent modification of histones, including acetylation, methylation, phosphorylation, ubiquitination, and sumoylation [64, 65]. Post-translational modifications of histones are facilitated by different enzymes.

Different histone modifications remodel the conformation of the chromatin, affecting the accessibility of transcription factors to a gene, and thereby regulating gene expression in a specific manner. Lysine residue acetylation and methylation are the most studied modifications. Histone acetylation of lysine residues in H3 and H4 tails catalyzed by histone acetyltransferases (HATs) has been consistently associated with active transcription in several studies [66, 67]. Deacetylation of histones by histone deacetylases (HDACs) correlates with DNA methylation and the inactive state of chromatin [39]. Histone methylation is also a major dynamic covalent epigenetic modification with more complex patterns. The lysine residue modification can be mono-, di-, or tri-methylated. Depending on the position in the histone chain, methylated lysines are associated with transcriptional activation or suppression. For example, H3K9 methylation state is strongly indicative of transcriptional repression and gene silencing, while H3K4 tri-methylation state is associated with gene activation [64, 68]. Histone methyltransferase (HMTs) catalyzes the transfer of a methyl group from SAM to a lysine residue either on H3 or H4, while histone demethylases eliminate methyl groups.

There has been a number of studies demonstrated that histone modification play a role in atherosclerosis [39, 69–71]. But limited evidence is available about the implication of HHcy in atherogenesis via histone modification. Since HHcy can inhibit SAM-dependent methyltransferases through elevated SAH, histone methylation might be influenced due to inhibited HMTs. In a recent study in rats, diet-induced HHcy was found to disturb global protein arginine methylation in a tissue-specific manner and affect H3 arginine 8 methylation in brain, along with reduced ADMA [72]. Consistently, in CBS-deficient mice, protein arginine hypomethylation was presented in liver and brain. ADMA of arginine 3 on H4 content was markedly decreased in liver [73]. Moreover, in the research to elucidate the role of the extracellular superoxide dismutase (EC-SOD) in the development of foam cells, accelerated DNA methylation of EC-SOD was induced by HHcy, as well as increased binding of acetylated H3 and H4 in momocytes [74]. Hcy-induced histone hyperacetylation was also observed in astrocytes [75].

### Therapy of hyperhomocysteinemia

The reduction in Hcy and the increased availability of methyl compounds provided by vitamin supplementation, such as folic acid, may not be sufficient to reverse epigenetic changes induced by HHcy [76]. It is possible that individuals with HHcy have an “Hcy memory effect” due to epigenetic alterations which continue to promote progression of cardiovascular complications even after Hcy levels are lowered. Deleterious effect of prior, extended exposure to elevated Hcy concentrations might have long-lasting effects on target organs and genes, hence underestimating the benefit of Hcy lowering therapies in CVD patients. Therapies targeting the epigenetic machinery as well as lowering circulating Hcy concentrations may have a more efficacious effect in reducing the incidence of cardiovascular complications.

## Conclusion

HHcy may be regarded as a global DNA hypomethylation effecter via SAH accumulation. While it is clear that epigenetic regulation involve in atherogenesis, it is unclear about the relative importance of global versus gene-specific methylation, nor is it clear how Hcy participates in the epigenetic modification. Global DNA hypomethylation may serve as a candidate mechanistic link between HHcy and atherosclerosis.

Further studies are warranted to unravel the mechanisms that select specific genes for epigenetic regulation in the presence of HHcy during atherogenetic process.

## Abbreviations

ASM: Allele-specific DNA methyation
meQTLs: Methlylation-associated loci
DNMT: DNA methyltransferase
CVD: Cardiovascular diseases
GWAS: Genome-wide association study
SNP: Single nucleotide polymorphism
HHcy: Hyperhomocysteinemia
Hcy: Homocysteine
SAM: S-adenosylmethionine
SAH: S-adenosylhomocysteine
MTHFR: 5,10-methyltetrahydrofolate reductase
CBS: Cystathionine β-synthase
MBD: Methyl-CpG-binding domain protein
SMC: Smooth muscle cells
VSMC: Vascular smooth muscle cell
ApoE: Apolipoprotein E
ER: Estrogen receptor
EC: Endothelial cell
DDAH: Dimethylarginine dimethylaminohydrolase
ADMA: Asymmetric dimethylarginine
eNOS: Endothelial nitric oxide synthase
HUVEC: Human umbilical vein endothelial cell
NO: Nitric oxide
TC: Total cholesterol
FC: Free cholesterol
CE: Cholesteryl ester
PPAR: Peroxisome proliferators-activated receptor
MCP-1: Monocyte chemoattractant protein-1
ABCA1: ATP-binding cassette transporter A1
ACAT1: Acyl-coenzyme A: cholesterol acyltransferase-1
HAT: Histone acetyltransferase
HDAC: Histone deacetylase
HMT: Histone methyltransferase
EC-SOD: Extracellular superoxide dismutase
BHMT: Betaine-homocysteine methyltransferase
DMG: Dimethylglycine
THF: Tetrahydrofolate.
